# Research progress on natural products in regulating the gut microbiota in Parkinson’s disease

**DOI:** 10.3389/fphar.2025.1667694

**Published:** 2025-09-08

**Authors:** Ying Jia, Yuanyuan Zhang, Xin Tai, Tengyu Zhao, Hanwen Zhang, Haichun Zhou

**Affiliations:** ^1^ The Second Clinical Medical College, Heilongjiang University of Chinese Medicine, Harbin, China; ^2^ The Fourth Affiliated Hospital of Heilongjiang University of Chinese Medicine, Harbin, China; ^3^ Medical Affairs Department, Heilongjiang Province Second Hospital, Harbin, China; ^4^ School of Basic Medicine, Heilongjiang University Of Chinese Medicine, Harbin, China

**Keywords:** Parkinson’s disease, natural product, gut microbiota, mechanisms, research progress

## Abstract

Parkinson’s disease (PD) is a progressive neurodegenerative disorder associated with α-synuclein deposition and dopaminergic neuron degeneration. Recent studies have revealed a close correlation between gut microbiota dysbiosis and the pathogenesis and progression of PD. Gut microbiota may influence the disease through multiple pathways, including promoting α-synuclein pathology, disrupting the gut-brain barrier, and triggering inflammation and neuronal damage. Currently, drug treatments for PD primarily focus on compensating for dopaminergic neurotransmission deficits, delaying neuronal degeneration, and clearing abnormal protein aggregates. However, these drugs can only slow disease progression and are associated with significant adverse effects. In contrast, natural products exhibit distinct advantages in modulating PD pathological features by targeting the “gut microbiota-metabolite axis,” owing to their multi-target synergistic regulation and favorable safety profiles, making them an ideal strategy for PD intervention. Based on this, we provide a comprehensive review of natural products that regulate the gut microbiota, analyze their specific mechanisms, and offer novel insights into this approach and provide a theoretical foundation for developing safe and effective PD therapeutics.

## 1 Introduction

Parkinson’s disease (PD) is the second most common progressive neurodegenerative disorder, with a prevalence of approximately 1%–2% among individuals aged 65 years or older, second only to Alzheimer’s disease. PD is more prevalent in males than females, and its global incidence continues to rise with the aging population, posing a pressing public health challenge (Ben-Shlomo et al., 2024). The clinical manifestations of PD primarily consist of characteristic motor symptoms. These motor symptoms include resting tremor, rigidity, bradykinesia, and postural instability. Additionally, PD is accompanied by various non-motor symptoms. A major manifestation of non-motor symptoms is gastrointestinal (GI) dysfunction, such as nausea, vomiting, gastroparesis, delayed gastric emptying, and constipation. Notably, a strong correlation exists between non-motor and motor symptoms. For instance, constipation affects up to 80% of PD patients and may precede motor symptoms by several years (Fasano et al., 2015). The pathophysiology of PD remains incompletely understood but is thought to involve multiple factors, including genetics, environmental influences, and lifestyle (Morris et al., 2024). Pathologically, PD is primarily characterized by the loss of dopaminergic neurons in the substantia nigra pars compacta (SNpc) and the abnormal aggregation of α-synuclein (α-syn). Recent studies suggest that pathological α-syn may originate in the GI tract, drawing attention to the potential link between gut microbiota and PD. This discovery has spurred the concept of the “gut-brain axis.” (Menozzi et al., 2025).

The gut microbiota (GM), the most complex microbial ecosystem in the human body, comprises trillions of microorganisms, including bacteria, fungi, viruses, and protozoa. It plays a pivotal role in health and disease and is often referred to as the “second brain.” Alterations in the diversity and abundance of GM have been implicated in the pathogenesis of various neurological disorders. Recent studies have demonstrated dysbiosis of GM in PD patients and animal models. Furthermore, fecal microbiota transplantation (FMT), probiotics, and prebiotics exhibit significant neuroprotective effects in PD (Tan et al., 2022). This indicates the ability of GM as a new target for PD intervention.

Natural products from natural sources exhibit properties such as water solubility, membrane permeability/transportability, biomolecular compatibility, and stability (Atanasov et al., 2021; Shenvi, 2024). They serve as a critical source for drug discovery, with 84% of clinically used central nervous system (CNS) drugs directly or indirectly originating from natural products. Remarkably, over 400 CNS drugs have been developed from just 20 natural products (Bharate et al., 2018). Bridging modern and traditional medicine, natural products have been extensively investigated for their therapeutic potential in PD. Numerous studies have demonstrated that natural products can modulate PD pathogenesis through multiple pathways, including anti-inflammatory and antioxidant effects. Their multi-target mechanisms and low toxicity profiles offer novel therapeutic avenues for PD management. Given these advantages, exploring natural products targeting the GM for PD treatment holds significant promise. This review summarizes several common classes of natural products with potential anti-PD effects, including polyphenols, flavonoids, polysaccharides, terpenoids and glycosides, and alkaloids. The aim is to provide a reference for the clinical application of GM-based natural product therapies in PD.

## 2 The relationship between gut microbiota and PD

### 2.1 Basic composition and function of gut microbiota

The microorganisms in the GI tract are predominantly bacteria, mainly strictly anaerobic bacteria (Zhernakova et al., 2016). The microbiota initially colonizes the human intestinal tract at birth, exhibiting maternal characteristics. The primitive microbiome (observed at approximately 1 year of age) is relatively simple but becomes increasingly complex during development due to environmental and dietary influences. The abundance and diversity of strict anaerobes have increased. These microbial communities persist throughout the host’s lifespan, maintaining relative stability despite being influenced by multiple factors (Milani et al., 2017). Notably, substantial interindividual variability exists in microbial composition among healthy subjects, particularly in infants, with gradual convergence toward similar phyla over time (Sorboni et al., 2022).

The GM comprises over 1,000 bacterial species (Hou et al., 2022). More than 90% of these are dominated by the phyla *Firmicutes* and *Bacteroidetes*, with lesser proportions of *Actinobacteria*, *Fusobacteria*, *Proteobacteria*, and *Verrucomicrobia* (Qin et al., 2010). The GM is characterized by high diversity, encompassing a vast array of microbial species and populations. This diversity maintains the equilibrium between microbes and the host, serving as a critical determinant of intestinal health (Li Z. et al., 2022). Existing in a symbiotic relationship with humans, the GM not only contributes to the regulation of GI and immune functions but also influences nutrient metabolism, metabolic regulation, neurofunctional modulation, as well as drug metabolism and absorption. It plays an essential role in maintaining host health and physiological functions, including those of the CNS.

### 2.2 Correlation between PD and gut microbiota

Disruption of GM homeostasis has been associated with various diseases, particularly PD. Alterations in microbial diversity and abundance have been observed in both animal models and human subjects. Through a systematic review and subgroup meta-analysis of 14 studies involving 1,045 PD patients and 821 healthy controls from different countries, Bai et al. demonstrated significant differences in GM abundance at the phylum, family, and genus levels between PD patients and healthy controls (Bai et al., 2024). Specifically, PD patients exhibited reduced abundance in four microbial families (*Lachnospiraceae*, *Prevotellaceae*, *Erysipelotrichaceae,* and *Faecalibacterium*) and increased abundance in six families (*Lactobacillaceae, Verrucomicrobiaceae, Bifidobacteriaceae, Rikenellaceae, Christensenellaceae,* and *Ruminococcaceae*). Heravi et al. conducted a systematic review of 26 PD studies and reported elevated abundance of *Akkermansia*, *Verrucomicrobiaceae, Lachnospiraceae,* and *Ruminococcaceae* in PD patients, whereas *Blautia, Coprococcus, Prevotellaceae,* and *Roseburia* were more abundant in controls (Heravi et al., 2023). Furthermore, GM composition in PD patients varies depending on geographical location and dietary habits (Shu et al., 2025). Microbial profiles also differ across PD stages: early-stage PD is characterized by dominant genera such as *Akkermansia, Alistipes, Anaerotruncus, Bilophila, Rikenellaceae, Verrucomicrobia,* and *Verrucomicrobiae*, whereas late-stage PD is associated with increased *Actinobacteriota* and *Erysipelotrichaceae* (Jiang et al., 2025). Similar microbiota alterations have been observed in non-human primate models of PD (Yan et al., 2021). These findings demonstrate that PD exhibits a distinct pattern of gut dysbiosis, which varies with disease progression and is influenced by geographical and dietary factors. This robustly supports the notion that GM dysregulation serves as a critical pathological feature and potential therapeutic target in PD.

The GM plays a pivotal role in the pathogenesis, progression, and severity of PD, exhibiting significant correlations with interindividual variability in clinical manifestations. It is associated not only with motor symptoms but also closely linked to non-motor impairments. *Bifidobacteriaceae* is strongly correlated with the severity of gait disturbances and the worsening of hallucinations (Zuo et al., 2022). The relative abundance of *Enterobacteriaceae* demonstrates a positive correlation with the severity of postural instability and gait difficulty (Scheperjans et al., 2015). In contrast, *Faecalibacterium* exhibits anti-inflammatory effects that confer protection to the intestinal epithelial barrier. A reduction in *Faecalibacterium* coupled with an increase in *Enterobacteriaceae* abundance may compromise the intestinal epithelial barrier, thereby rendering the enteric nervous system (ENS) more susceptible to luminal pathogens. *Prevotellaceae* is associated with the severity of PD. Its reduction correlates with decreased secretion of ghrelin (Silva-Reis et al., 2022). A negative correlation exists between the abundance of *Lachnospiraceae* and PD duration. Meanwhile, *Lachnospiraceae* can contribute to GI dysmotility in PD patients (Unger et al., 2016). *Lactobacillaceae* may exacerbate motor dysfunction (van der Maden et al., 2025). Barichella’s study (Barichella et al., 2019) demonstrated that alterations in the abundance of *Lactobacillaceae* (increased) and *Lachnospiraceae* (decreased) constitute the most profound factors associated with PD clinical features (cognitive impairment, gait disturbance, and postural instability). Notably, *Lachnospiraceae* was significantly more abundant in patients with the non-tremor-dominant phenotype. Mehanna et al. (2023) reported reduced *Bifidobacterium* levels in the GM of tremor-dominant PD patients, decreased *Lactobacillus* in non-tremor-dominant phenotypes, and a marked increase in *Bacteroides* among mixed phenotypes. A prospective study by Cilia et al. (2021) involving 39 early-stage PD patients revealed that the abundance of *Ruminococcaceae* and *Actinobacteria* correlated with accelerated decline in global cognitive function. The composition of the GM is significantly associated with the onset, progression, severity, and diverse clinical manifestations of PD. Changes in the abundance of specific bacterial families directly impact the pathological processes and individualized clinical presentations of PD.

### 2.3 Microbial metabolites in PD

Microbial metabolites act as pivotal mediators within the microbiota-gut-brain axis (MGBA) (Zheng et al., 2021). These metabolites are broadly classified into three categories: (1) Diet-derived metabolites produced directly by microbial digestion, such as short-chain fatty acids (SCFAs) and indole derivatives; (2) *De novo* synthesized metabolites originating from the microbiota; (3) Host- or diet-derived compounds modified by microbial activity.

#### 2.3.1 SCFAs

The production of SCFAs is primarily mediated by the fermentation of carbohydrates and dietary proteins by *Bifidobacterium*, *Lactobacillus*, *Faecalibacterium*, *Lachnospiraceae*, and *Ruminococcaceae*. SCFAs are a group of organic fatty acids, predominantly comprising acetate, propionate, butyrate, and β-hydroxybutyrate. SCFAs play a profound role in maintaining intestinal homeostasis, modulating metabolism, regulating immune function, and influencing neurological activity (Dalile et al., 2019; Qu et al., 2025). Notably, the concentration of SCFAs is significantly reduced in patients with PD (Unger et al., 2016). Butyrate not only acts on the colonic mucosa, but also induces hepcidin expression to maintain iron homeostasis and mitigates neuronal ferroptosis (Mayneris-Perxachs et al., 2022). As a histone deacetylase (HDAC) inhibitor, sodium butyrate regulates gene expression, protects dopaminergic neurons, and prevents motor deficits in PD models (Wang F. et al., 2024; Zhang L. Y. et al., 2024). Propionate modulates energy metabolism in the gut and exerts neuroprotective effects via choline-neuronal signaling. Additionally, it suppresses neurodegeneration through nonautonomous mechanisms (Wang C. et al., 2024).

#### 2.3.2 Bile acids

Primary BAs are predominantly synthesized in the liver from cholesterol and include free bile acids (CA and CDCA), as well as their glycine (Gly)- or taurine (Tau)-conjugated forms, which exhibit enhanced water solubility (GCA, TCA, GCDCA, and TUDCA). Approximately 95% of BAs released into the large intestine via the gallbladder are reabsorbed into the liver through the ileal apical sodium-dependent bile acid transporter (ASBT). A minor fraction undergoes deconjugation and dehydroxylation by GM (e.g., *Lactobacillus*, *Bifidobacterium*, *Bacteroides*, *Clostridium*) possessing bile salt hydrolase (BSH) activity, thereby converting them into secondary BAs such as LCA, DCA, and UDCA (Wang S. et al., 2023).

An increased abundance of BA-synthesizing bacteria (*Burkholderiales* and *Clostridium*) was observed in the appendix tissues of PD patients, accompanied by significantly elevated levels of LCA and DCA in the ileum (Li P. et al., 2021). In a prospective study investigating the relationship between PD and plasma microbial metabolites, Zhao et al. identified altered abundances of CDCA and GCA, which contrasts with previous reports of elevated secondary BAs levels in PD patients’ blood samples (Zhao et al., 2025).

#### 2.3.3 Metabolic product of tryptophan

##### 2.3.3.1 Indoles and derivatives

Tryptophan, the sole essential amino acid containing an indole structure, is primarily obtained from protein-rich foods. In the gut, it is metabolized through three major pathways—the indole pathway, kynurenine (Kyn) pathway, and serotonin pathway—with the GM playing a pivotal role in each. Particularly in the indole pathway, GM drive the catabolism of tryptophan into indole and its derivatives. The structural diversity of indole derivatives arises from variations in bacterial-encoded glycosidases. Over 85 bacterial species, including *Escherichia coli*, *Bacteroides*, and *Clostridium*, produce indole; *Clostridium* generates indole-3-propionic acid (IPA) and tryptamine; *Lactobacillus* synthesizes indole-3-lactic acid (ILA) and indole-3-aldehyde (IAld); *Peptostreptococcus* produces indole-3-acrylic acid (IA); and *Ruminococcus* and *Clostridium* yield tryptamine, among others. As a class of profoundly bioactive molecules, indole and its derivatives play a complex and critical role in the pathological mechanisms and therapeutic research of PD. Their functions span multiple dimensions, including GM metabolism, intestinal epithelial barrier integrity, mitochondrial function regulation, neuroinflammatory modulation, neuroprotection of dopaminergic neurons, and α-syn pathological aggregation (Zhou et al., 2023).

Shao’s study demonstrated that plasma ILA levels were reduced in PD patients (Shao et al., 2021), accompanied by decreased abundance of ILA-producing *Clostridium saccharolyticum* in fecal samples. Additionally, alterations were observed in several other *Clostridium* and *Bacteroides* species involved in tryptophan metabolic enzymes (Bedarf et al., 2017). Chen’s investigation of 56 PD patients and 43 age-, sex-, and diet-matched healthy controls (family members or friends) revealed unchanged plasma tryptophan levels but significantly elevated IPA concentrations in PD patients (Chen S. J. et al., 2022). IPA functions as a HDAC inhibitor and exhibits positive correlation with pathological protein aggregation.

##### 2.3.3.2 5-HT and kynurenine

5-HT is synthesized from its precursor tryptophan and is widely distributed in both the central and peripheral nervous systems. The intestine serves as the primary site (accounting for ∼95%) of peripheral 5-HT synthesis. Most 5-HT is released from enterochromaffin cells (ECCs), which directly interact with enteric nerve terminals, playing a pivotal role in normal intestinal function. Through Mendelian randomization analysis of the GM-PD relationship, Li et al. demonstrated that intestinally derived 5-HT is associated with an earlier age of PD onset (Jiang et al., 2023). Postmortem studies of PD patients revealed elevated 5-HT1A receptor availability in the anterior cingulate cortex (Gonzaga et al., 2025). The Kyn pathway, representing the dominant route (≥95%) of tryptophan metabolism, exhibits broad pleiotropic bioactivities including neuroprotective/neurotoxic effects. Its directional shift profoundly impacts neuronal function and survival. Metabolomic analyses by Chang et al. identified a transition from neuroprotective kynurenic acid (KA) to neurotoxic quinolinic acid (QA) in PD patients (Chang et al., 2018). Serum KYN levels were significantly reduced in PD patients, accompanied by decreased KA/KYN conversion rates and substantial alterations in tryptophan metabolic pathways (Fan et al., 2024). The GM suppresses Kyn production and promotes 5-HT synthesis. *Akkermansia* modulates tryptophan metabolism, thereby shifting metabolic flux from Kyn to 5-HT and enhancing 5-HT activity (Wang J. et al., 2021; Zhang L. et al., 2023; Pan et al., 2025).

#### 2.3.4 LPS

Lipopolysaccharide (LPS), a major component of the outer membrane of Gram-negative bacteria. The intestine serves as the primary source of LPS. Under physiological conditions, a healthy intestinal barrier (IB) confines the majority of LPS within the intestinal lumen. However, when the IB is compromised or dysbiosis occurs, LPS translocates into the bloodstream in large quantities, entering the portal circulation and triggering metabolic endotoxemia (Popovic et al., 2023; Gorecki et al., 2025). LPS and its associated inflammatory response compromise the blood-brain barrier (BBB), increasing permeability (Ma and Lieberman, 2024). This enables the translocation of LPS and inflammatory mediators into the CNS, subsequently activating microglia and astrocytes. Such activation propagates a neuroinflammatory cascade that promotes α-syn pathology, impairs dopaminergic neurons, and disrupts neuronal homeostasis (Huang et al., 2024). Additionally, LPS exerts direct neurotoxicity, inducing neuronal apoptosis or necrosis.

In patients with PD, the abundance of Gram-negative bacteria producing LPS is elevated, and this abundance positively correlates with the severity of motor symptoms. Studies have demonstrated increased levels of LPS-binding protein and soluble CD14 (proteins associated with the LPS/TLR4 signaling pathway) in PD patients, which correlate with disease progression. Administration of LPS into the SN or the GI tract of rodents successfully induces PD-like motor symptoms and pathological changes (Gorecki et al., 2019).

#### 2.3.5 Gasotransmitters

Gasotransmitters, primarily produced by the GM, serve as pivotal signaling mediators and effector molecules *in vivo*. *Sulfate-reducing bacteria* generate substantial amounts of hydrogen sulfide (H_2_S), while other bacteria (e.g., certain *Clostridium* and *Bacteroide*) also produce H_2_S via enzymatic reactions. *Lactobacillus*, *Bifidobacterium*, and *E*.*coli* contribute to nitric oxide (NO) production. H_2_S exhibits a dual role. At physiological concentrations, it demonstrates neuroprotective properties, such as suppressing reactive oxygen species (ROS) generation, inhibiting microglia (MG) activation, and restoring mitochondrial function (Wang L. et al., 2023). Conversely, excessive H_2_S elevates ROS levels, disrupts barrier integrity, triggers inflammation, and promotes α-Syn aggregation (Murros, 2022). A meta-analysis revealed a significant increase in H_2_S-producing bacteria in PD patients (Marzouk et al., 2025). Similarly, NO concentration critically determines whether it induces or suppresses inducible nitric oxide synthase (iNOS) expression. iNOS participates in defense mechanisms involving immune cells and oxidative responses (Picón-Pagès et al., 2019).

### 2.4 Pathogenic mechanisms of gut microbiota in PD

**FIGURE 1:**
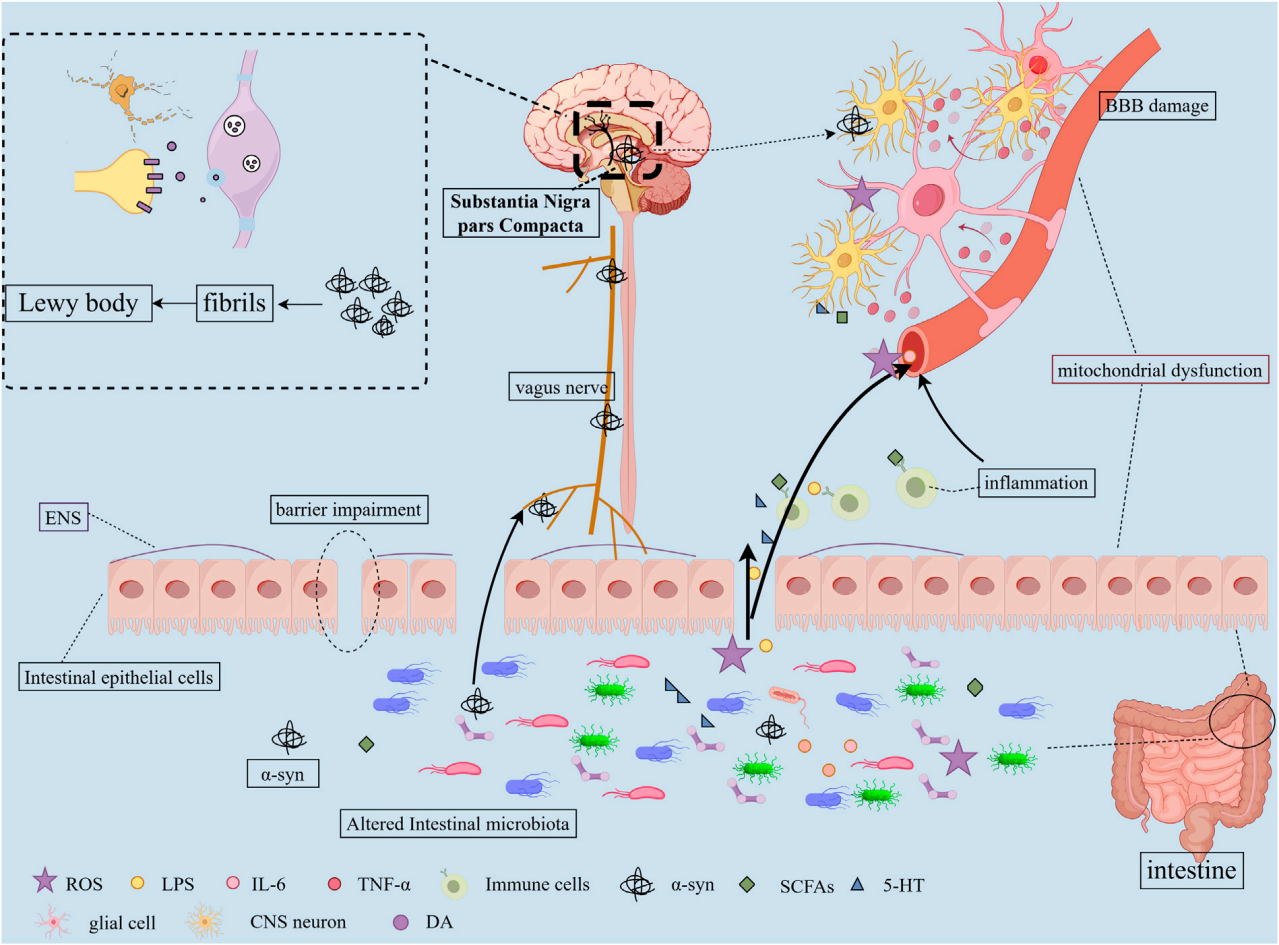
Pathogenic Mechanisms of Gut Microbiota in PD. 1) Dysbiosis of the GM alters microbial metabolites and compromises the intestinal barrier function, resulting in “leaky gut” syndrome; 2) These metabolites and harmful substances enter systemic circulation, damaging BBB structure and function while activating glial cells and triggering neuroinflammation; 3) Gut-derived α-syn may propagate to the CNS via vagal nerve pathways, ultimately forming fibrillary aggregates in the SNpc; 4) Homeostatic imbalance induces chronic low-grade inflammatory states in both CNS and peripheral systems; 5) Microbial-derived toxins contribute to mitochondrial dysfunction; 6) These cascades ultimately lead to dopaminergic neuron loss in the SN and α-syn aggregation.

Mounting evidence indicates the existence of a bidirectional gut-brain communication system between the CNS and the GM, termed the MGBA (Ohara and Hsiao, 2025). The microbial community can interact with the immune system, neuroendocrine pathways, and the sympathetic nervous systems, thereby modulating brain function, ultimately contributing to the pathogenesis and progression of PD (Zhang X. et al., 2023). Conversely, the CNS can induce alterations in intestinal motility, secretion, permeability, and inflammation through autonomic interactions. The specific mechanism diagram is shown in the figure (Figure1).

#### 2.4.1 Pathological α-synuclein production

α-Syn is primarily localized to presynaptic terminals in the CNS. Under physiological conditions, α-syn exists in a dynamic equilibrium between unfolded monomers and α-helix-folded tetramers, exhibiting low aggregation propensity and playing a crucial role in neurotransmission (Lashuel et al., 2013). Under pathological conditions, misfolded α-syn aggregates and further matures into fibrillar oligomers, disrupting normal cellular processes and contributing to neuronal death. Notably, phosphorylated α-syn levels were found to be significantly elevated in the GI up to 20 years before PD diagnosis (Hilton et al., 2014).

The GM is closely associated with pathological α-syn (Fang et al., 2024). In an eQTL analysis, it was found that the increased abundance of opportunistic bacteria in the intestines of PD patients was directly correlated with genetic variations in SNCA, the gene encoding α-syn (Wallen et al., 2021). α-syn-overexpressing (ASO) mice treated with broad-spectrum antibiotics did not develop motor dysfunction or exhibit α-syn aggregates in the brain. Furthermore, FMT from healthy donors ameliorated motor deficits in ASO mice, whereas colonization with microbiota from PD patients exacerbated the impairments (Sampson et al., 2016). Extracellular vesicles secreted by *E*.*coli* can deliver curli to colonic mucosal epithelial cells, upregulating DAPK1 expression and triggering α-syn aggregation (Liang et al., 2023). *Enterobacteriaceae* promote nitrite production. This mediates the oxidation of Fe^2+^ to Fe^3+^ and facilitates dopamine oxidation to o-quinone. Consequently, these processes induce misfolding and aggregation of α-syn in enteroendocrine cells (Ortiz de Ora et al., 2024). HpmA derived from *Proteus mirabilis* contributes to α-syn oligomerization and membrane pore formation (Huh et al., 2023a). *Akkermansia muciniphila* induces α-syn aggregation in enteroendocrine cells (EECs) (Amorim Neto et al., 2022).

Both LCA and DCA were found to markedly accelerate α-syn aggregation, exacerbating neurotoxicity while reducing the lag phase by 75% and 30%, respectively (Kaur et al., 2024). CDCA effectively reduces α-syn expression in PD model mice. Additionally, it alleviates motor deficits and anxiety-like behaviors in these models (Mehreen et al., 2025). Overexpression of α-syn leads to age-dependent dysregulation of the GM (Singh et al., 2023). Animal studies further demonstrate that α-syn drives ecological imbalance in the murine microbiome (Sampson et al., 2025).

#### 2.4.2 Impairment of barrier function

The intestinal barrier (IB) and blood-brain barrier (BBB) are critical physiological barriers that maintain microenvironmental homeostasis (Aburto and Cryan, 2024). Dysfunctions of both barriers occur in PD patients and animal models. In PD patients, colonic ZO-1 expression is reduced (Liao et al., 2024). A PD monkey model shows significantly decreased occludin and ZO-1 expression in the duodenum, ileum, colon, and rectum, with concurrent intestinal mucosal damage (Zhang Y. et al., 2025). Postmortem PD studies reveal vascular changes and increased BBB permeability (Lau et al., 2024). Notably, Elabi et al. first observed impaired BBB integrity in an ASO mouse model. This impairment features increased extravascular fibrinogen, pericyte activation, and basilar membrane Col IV reduction (Elabi et al., 2021).

GM and metabolites modulate both IB and BBB functions. Germ-free mice exhibit enhanced BBB permeability and impaired tight junctions (Braniste et al., 2014). *Lactobacillus rhamnosus* (with strong adhesive properties) physically regulates goblet cells and mucus layers, restoring IB function via anti-inflammatory and cytoprotective effects (Martín et al., 2019). Propionate reverses antibiotic-induced BBB permeability and protects against BBB oxidative stress (Hoyles et al., 2018; Chenghan et al., 2025). It also upregulates tight junction proteins via AKT signaling, improving intestinal barrier function and motility (Huang et al., 2021). Additionally, propionate may exert neuroprotection through the FFAR3-GLP-1 axis in enteric neurons (Hou et al., 2021). Butyrate significantly increases occludin and claudin-5 expression in colonic tissue and the prefrontal cortex, repairing IB and BBB damage (Wang et al., 2025d). LPS induces pyroptosis in brain endothelial cells via the Casp11/Cd14-GSDMD pathway, causing BBB ultrastructural changes (Wei et al., 2024; Wei et al., 2025). Gut-derived α-syn impairs IB through caspase-1 inflammasome signaling and disrupts BBB via pericyte interactions (Dohgu et al., 2019; Pellegrini et al., 2022). IPA enhances IB by 1) reducing paracellular permeability by increasing transepithelial electrical resistance; 2) promoting mucin/goblet cell secretion; and 3) thickening mucus layers (Li J. et al., 2021). IAA activates AHR to promote goblet cell differentiation and mucus secretion (Cui W. et al., 2024). Indole supplementation repairs barrier damage in tryptophan-deficient mice. Tryptamine increases colonic ion flux and fluid secretion. This is supported by accelerated GI transit in germ-free mice colonized with tryptamine-producing engineered *Bacteroides* (Bhattarai et al., 2018). 5-HT regulates intestinal stem cells and mitigates IB damage via the Htr4-Kit-Wnt3 axis (Zhang D. et al., 2025).

#### 2.4.3 Immunomodulation

The immune response drives PD pathophysiology. GM alterations trigger intestinal inflammation, damage the IB, and activate mucosal immune cells. These changes promote pro-inflammatory factor release alongside α-syn misfolding/aggregation. Such pathological products circulate to the brain, activating central immune cells and inducing neuroinflammation. Ultimately, this cascade causes dopaminergic neuron loss and PD progression. In PD patients, CD3^+^ T cell density in the SNpc negatively correlates with disease duration. Iba-1 expression is significantly elevated (Backman et al., 2025). Microglial and astrocyte activation is prominent (Kam et al., 2020). Peripheral T lymphocytes show increased PD-1 expression, which positively correlates with IFN-γ levels in CD4^+^ T cells (Zhang Z. L. et al., 2025). CD4^+^ T cell infiltration rises in cerebrospinal fluid and SNpc (observed in both patients and animal models) (Sun et al., 2024b). Clinical evidence further links gut immunity to PD. Chronic appendicitis-like lesions occur in 53% of PD patients (Chen et al., 2021). Appendectomy reduces PD risk (Nakahara et al., 2023). Inflammatory bowel disease (IBD) increases PD risk (Espinosa-Oliva et al., 2024; Kars et al., 2024). Anti-TNF-α therapy in IBD patients lowers PD risk by 78% (Peter et al., 2018).

Altered GM frequently coincides with elevated inflammatory cytokines. Specifically, *Verrucomicrobia* abundance correlates with plasma IFN-γ levels, whereas *Bacteroides* associates with TNF-α concentrations (Lin et al., 2019). *Akkermansia muciniphila* modulates SCFAs, ameliorating neuroinflammation and promoting hippocampal neurogenesis in PD models (Huang, 2024). *Alistipes* colonization regulates lipid metabolism and suppresses inflammatory mediators via outer membrane vesicles (Older et al., 2025). LPS crosses the BBB, activating MG through TLR4/MyD88/NF-κB signaling. FMT counteracts this by reducing LPS levels and inhibiting TLR4/MyD88/NF-κB in both gut and brain, attenuating inflammation (Zhao et al., 2021). Notably, LPS-stimulated macrophages secrete TNF-α, correlating with motor symptom severity. Concurrently, SCFAs associate with CD3^+^ T cells and TLR4^+^ cells while downregulating regulatory T cells (Perez-Pardo et al., 2019). Butyrate activates FFAR3, modulating sympathetic signaling and enteric neurogenesis. This consequently influences energy metabolism and inflammatory responses (Kalkan et al., 2025). BA mediate gut-brain communication via the FXR/TGR5-GLP-1 and FXR-FGF15/19 pathways, exerting neuroprotective effects (Kiriyama and Nochi, 2019). TUDCA activates TGR5, regulating AKT/NF-κB, AMPK/mTOR, and Pink1/Parkin pathways to suppress microglial activation and enhance autophagy (Ni et al., 2025). Indole derivatives act as core AhR ligands. AhR activation enhances intestinal barrier function, promotes IL-22 secretion, inhibits M1 macrophage polarization, and modulates gut immunity (Ma et al., 2020). Paradoxically, it may disrupt BBB integrity and exacerbate neuroinflammation (Salminen, 2023). IPA suppresses NF-κB via the PXR, reducing proinflammatory factors while exhibiting antioxidant activity (Zhao et al., 2022; Niu et al., 2025). Furthermore, IPA ameliorates dopaminergic dysfunction by inhibiting the IL-13Rα/JAK1-STAT6 signaling and reducing enteric glial cell gliosis (Shang et al., 2025).

#### 2.4.4 Vagus nerve

The VN serves as the primary neural pathway connecting the gut and the brain, representing the fastest and most direct route. Although the VN is widely distributed throughout the gastrointestinal tract, it does not directly interact with the GM. Instead, communication occurs through specialized structures called neuropods on enteroendocrine cells (Peterson, 2020).

Epidemiological and histopathological findings suggest that pathological α-Syn may propagate from the gut to the brain via the VN, thereby increasing the risk of PD (Braak et al., 2003; Kim et al., 2019; Borghammer et al., 2022; Yang Z. X. et al., 2024). In mice inoculated with α-Syn PFFs, Lewy body (LB)-like aggregates formed in the dorsal motor nucleus of the VN and the myenteric plexus at 45 days and 12 months post-inoculation. However, these aggregates were completely absent when the VN was severed prior to inoculation (Uemura et al., 2019). Regarding the mechanism of α-Syn transport to the VN, Rashmi Chandra et al. provided evidence that intestinal mucosal cells facilitate this translocation (Chandra et al., 2023). High-resolution ultrasonography has revealed VN atrophy in PD patients (Dong S. et al., 2024). VN stimulation, as an adjunctive therapy, has been shown to ameliorate PD-related pathology and motor dysfunction (Evancho et al., 2024; Zhang H. et al., 2025).

*Enterococcus faecalis* can influence brain function in PD mice via the vagal pathway, mitigating BBB damage and neuroinflammation. However, this protective effect is attenuated upon subdiaphragmatic vagotomy (Shao et al., 2025). *Lactobacillus* rhamnosus modulates the expression of GABA receptors in the mouse brain and affects neuro-emotional behaviors, yet these changes are blocked by vagotomy (Bravo et al., 2011). LPS can activate vagal afferents through TLR4 on the nodose ganglion (Hosoi et al., 2005). SCFAs directly stimulate vagal afferent activity (Pang et al., 2026).

#### 2.4.5 Mitochondrial dysfunction and oxidative stress

Mitochondrial dysfunction is a central mechanism in PD pathogenesis. As critical energy suppliers, mitochondria sustain dopaminergic neuron function. Impairment of the mitochondrial electron transport chain (ETC.) elevates ROS production. Excessive ROS damages mitochondrial components—including membrane lipids, proteins, and DNA (mtDNA)—compromising mitochondrial integrity. Concurrently, ROS attacks nuclear DNA, causing widespread cellular damage (Islam et al., 2018). Elevated oxidative stress markers confirm this pathology in PD patients (Korczowska-Łącka et al., 2023). Mitochondria also regulate Ca^2+^ uptake to maintain neuronal calcium homeostasis. Mitochondrial quality control (MQC) mechanisms sustain functional mitochondria through mitophagy, fusion/fission dynamics, and biogenesis. Dysregulation of these mechanisms increases ROS and reduces ATP. Notably, mutations in *PINK1* and *Parkin*—key autophagy genes—are linked to early-onset PD (Samaranch et al., 2010). Reduced nigral PGC-1α expression in PD contrasts with its neuroprotective effects when activated (Mohammed et al., 2024; Ma et al., 2025). Both sporadic and familial PD exhibit mitochondrial dysfunction (Banerjee et al., 2009). Aberrant expression of mitochondrial-associated genes elevates PD risk (Wang et al., 2025e). Therapeutic targeting of oxidative stress and mitochondrial defects shows neuroprotective efficacy in PD models (Li X. et al., 2024). Clinically, blood mitochondrial DNA copy number (mtDNA-CN) may serve as a diagnostic and prognostic biomarker (Jo et al., 2025).

GM couples with mitochondrial dynamics (Zhu et al., 2022). SCFAs traverse the BBB, modulating mitochondrial metabolism (Chen and Vitetta, 2020). Butyrate curbs ROS/RNS in immune cells (Kalyanaraman et al., 2024); propionate reinforces mitochondrial integrity in Tregs (Duscha et al., 2020). LPS drives mitochondrial ROS accumulation, fueling inflammation. *B. animalis* ssp. Activates nigral PGC-1α to suppress neuroinflammation (Dong Y. et al., 2024). Microbial toxins target mitochondria: *Clostridium*’s TcdB inhibits mitoK^+^ channels (apoptosis induction) (Matarrese et al., 2007); *H. pylori* VacA triggers mitochondrial fission (Foo et al., 2010). Pathogenic α-syn disrupts mitochondria by:1) Inner membrane binding that inhibits MC I, driving dysfunction and mitophagy in A53T models; 3) disrupting morphology through autophagic flux in SH-SY5Y cells (Plotegher et al., 2014; Du et al., 2020). α-Syn PFFs induce dopaminergic oxidative stress via p-NMDAR2B/Nur77 (Lai et al., 2025). 5-HT elevates antioxidant defenses, reduces ROS, and enhances mitobiogenesis via SIRT1-PGC-1α (Fanibunda et al., 2019).

## 3 The related natural products regulating the PD gut microbiota

**FIGURE 2:**
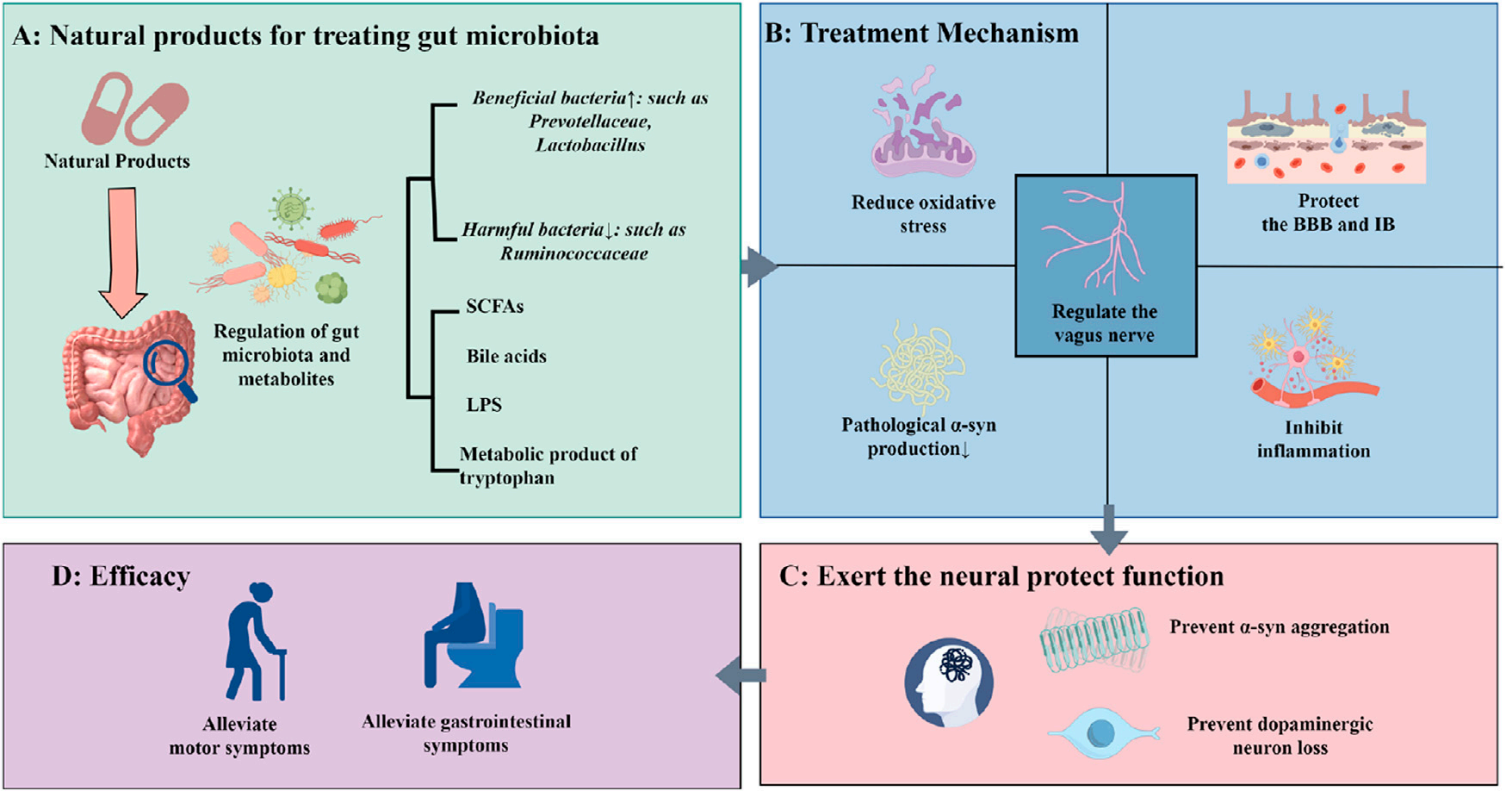
Natural Products Delay the Progression of PD via Gut Microbiota. **(A)** Regulation of gut microbiota and microbial metabolites by natural products; **(B)** Therapeutic mechanisms of natural products in PD treatment through gut microbiota modulation; **(C)** Neuroprotective effects mediated by attenuation of pathological changes in the PD brain; **(D)** Improvement of clinical PD symptoms by natural products.

**FIGURE 3:**
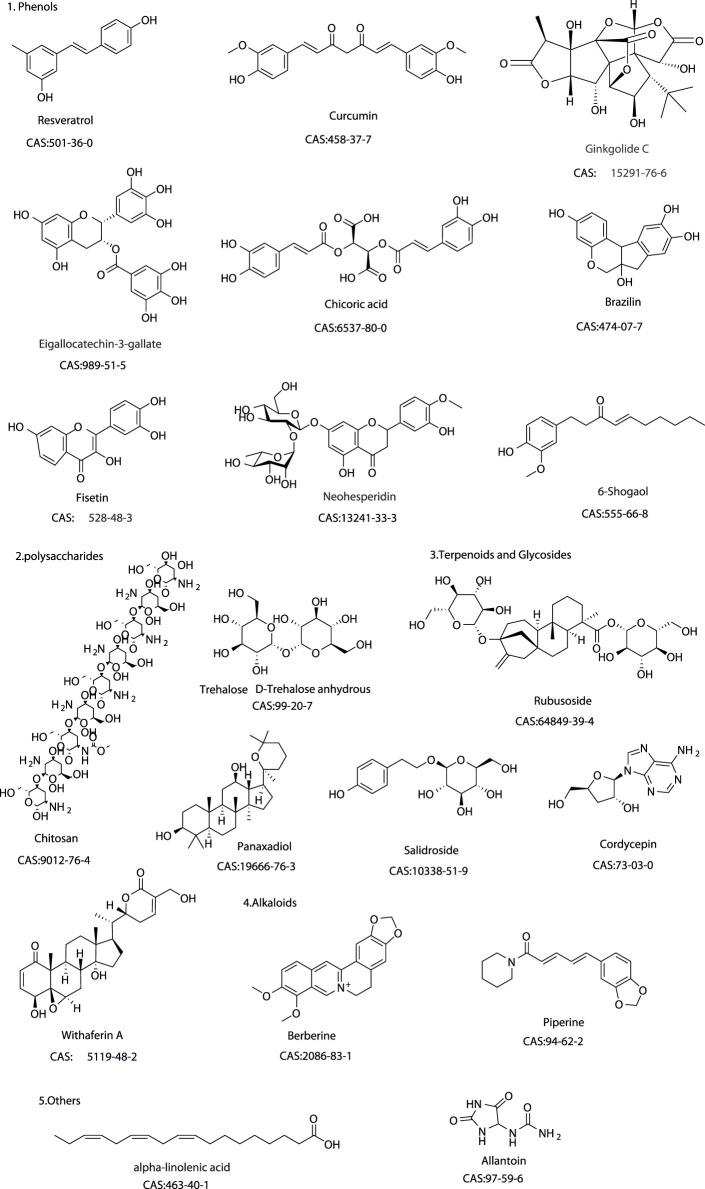
Chemical structure of these natural products.

This review first delineates the most recent insights into the role of GM dysbiosis and the gut-brain axis in the pathogenesis of PD. Subsequently, we summarize and analyze experimental studies, and clinical evidence concerning the application of natural products for PD treatment via GM modulation over the past decade (Table 1). We synthesize the efficacy and underlying mechanisms by which these natural products ameliorate PD symptoms through restoring gut microbial balance and influencing gut-brain communication pathways (Figure 2). Representative chemical structures of key bioactive natural compounds are illustrated in Figure 3.

**TABLE 1:** Summary of recent studies on the Related Natural Products Regulating the Gut Microbiota in PD.

Classification	Natural products	Source	Experimental subjects	Changes in gut microbiota	Possible mechanisms	References
Phenols	Resveratrol	Jackfruit, Grapes, Red wine	MPTP/P-induced c57 mice	*Prevotellaceae*, *Rikenellaceae*, *Erysipelotrichaceae*, *Blautia*, *Alistipe*↑ The ratio of *Fimicutes*/*Bacteroidetes* and *Lachnospiraceae*, *Akkermansia*↓	Inflammation↓ TH-positive cells in the SNpc and TH-positive fiber density in the striatum↑	Tao et al. (2023)
			A53T mice	*Lactobacillus murinus*, *Lactobacillus reuteri*, *Enterorhabduscaecimuris*, *Lactobacillus Taiwanensis*, *Lactobacillus*↑	Abnormal amino acid metabolism↓ Mitochondrial dysfunction↓ Oxidative stress↓ Neuroinflammation↓	Sun et al. (2024a)
			MPTP/P-induced c57 mice	--	TLR4/MyD 88/NF-κB pathway↓	Gui et al. (2024)
	Curcumin	Curcuma	MPTP-induced c57 mice	Modulating the gut microbiota	NF-κB and NLRP3 pathways↓ Neuroinflammation↓	Li et al. (2025a)
			MPTP-induced c57 mice	*Muribaculaceae*, *Lactobacillaceae*, *Lachnospiraceae*, *Eggerthellaceae*↑ *Aerococcaceae*, *Staphylococcaceae*↓	Glial cell activation nd Neuroinflammation↓ Tyrosine and levodopa↑ Neuroprotection of the gut flora-metabolite axis↑	Cui et al. (2022)
			MPTP-induced c57 mice	The *Firmicutes*/*Bacteroidetes* ratio↓ *Turicimonas* and *Culturomica*↑ *Desulfovibrio*, *Mucispirillum* and *Schaedlerella*↓	Regulate carbohydrate metabolism by directly altering the concentration and proportion of SCFAs. Gastrointestinal dysfunction↓ Dopaminergic neurons↑ Intestinal barrier dysfunction↓	Cai et al. (2023)
	Chicoric acid	Chicory, Dandelion, and Echinacea	MPTP-induced c57 mice	Phylum *Firmicutes*, genera *Lactobacillus* and *Ruminiclostridium*↑ Phylum *Bacteroidetes* and genera *Parabacteroide*↓	The TLR4/MyD88/NF-κB signaling pathway↓ Inflammation↓ Glial hyperactivation↓	Wang et al. (2022a)
	Eigallocatechin-3-gallate	Green tea	The *Drosophila melanogaster* with PINK1 mutations	At the phylum level, *Proteobacteria*↓ *Firmicutes* and *Bacteroidetes*↑ At the genus level, *Acetobacter* and *Lactobacillus*↓ Some taxa of interest, such as *Phascolartobacterium*, *Clostridiales*, *Faecalibacterium*↑	The GM-TotM pathway↑ Neuroprotection↑	Xu et al. (2020)
	Typha pollen flavonoids	Typha angustifolia	MPTP-induced c57 mice	*Muribaculum*, *Turicimonas*, *Bacteroides*, *Muribaculaceae*, *Rodentibacter* and *Parabacteroides*↑ *Proteus*, *Helicobacter*, *Firmicutes*, *Acetatifactor*, *MucIspirillum* and *Acutalibacter*↓	Oxidative stress↓ AMPK-Related Pathways↑ Ferroptosis Pathways↓	Wang (2024)
	Anthocyanin	Flowers, Fruits, Stems, Leaves, and roots of higher plants	MPTP-induced c57 mice	*Bacteroidetes* phylum and *Alistipes* genus↑ *Patescibacteria* phylum↓	Inflammation↓	Wang et al. (2025c)
			MPTP-induced c57 mice	*Norank_f__Muribaculaceae, Coriobacteriaceae_UCG-002* and *Parvibacter*↓ *Norank_f__Eubacterium_coprostanoligenes_group*↑	*Coriobacteriaceae_UCG-002* and glycerophospholipid metabolic pathways	Cao et al. (2024)
			Gut microbiota of healthy individuals	The F/B ratio↓ Beneficial bacteria (*Parabacteroides*, *Bifidobacterium*, *Lachnospira*, *Dialister*, *Blautia*)↑ Actinobacter↑ Harnful bacteria↓	Adjust the microbial community structure	Gao (2022)
			Gut microbiota of healthy individuals	*Bacteroidetes*↓ *Firmicutes* and *Verrucomicrobia*↑	Lipid metabolism Amino acid metabolism Neurodegeneration↓ Intestinal Barrier Destruction↓	Wang et al. (2022c)
	Daidzein	Soybeans	6-OHDA and MPP^+^-induced SH-SY 5 Y cells	The gut microbiota converts Daidzein into Equol	Cytotoxicity↓ TLLR4/MAPK/NF-κB signaling pathway↓	Johnson et al. (2020)
	Ginkgolide C	Ginkgo biloba leaves	MPTP-induced c57 mice	At the phylum level,The F/B ratio, *Fusobacteria*↓ *Actinobacteriota* and *Proteobacteria*↑ At the family level, *Erysipelotrichaceae*, and *Paraprevotellaceae*↓ *Coriobacteriaceae*↑ At the genus level, *Allobaculum*↓ *Adlercreutzia*, *Lactobacillus*, and *Flexispira*↑	The AKT/Nrf 2/HO-1 pathway The NF-κB and MAPK pathway↓	Gao et al. (2024)
	Brazilin	Caesalpinia sappan L.	MPTP-induced c57 mice	*Firmicutes*↑ *Bacteroidetes*↓	Oxidative stress and Neuroinflammation↓ Repaire the intestinal barrier	Gao et al. (2025)
	Neohesperidin	Poncirus and Citrus	MPTP-induced c57 mice	At the phylum level, *Bacteroidetes*↑ *Actinobacteria*, *Proteobacteria*↓ At the family level, *Lactobacillaceae*,*S24-7*↑ *Erysipelotrichaceae*, *Desulfovibrionaceae*, *Ruminococcaceae*↓ At the genus level, *Lactobacillus*↑ *Adlercreutzia*, *Allobaculum*, *Oscillospira*, *Psychrobacter*↓	Neuroinflammation↓ The NF-κB and MAPK pathway↓	He et al. (2024)
	Fisetin	Strawberries, apples	MPTP-induced c57 mice	*Lachnospiraceae*↑ *Uncultured_bacterium_g_Escherichia-Shigella* and u*ncultured_bacterium_g_Bacillus*↓	Neuroprotection↑	Chen et al. (2020)
	6-Shogaol	Zingiber officinale	MPTP-induced c57 mice	Microbiome-induced abnormalities↓	Neuroinflammation and apoptosis↓ The intestinal barrier dysfunction↓	Huh et al. (2020)
Polysaccharides	Chitosan	Crustaceans and mollusks	MPTP-induced c57 mice	Gut flora diversity↓	Acetate levels↓ Inflammation by the PPARD/AMPK signaling↓ The intestinal barrier and BBB disruption↓	Wang et al. (2026)
	Fucoidan	Brown algae	ROT-induced PD mice	*Akkermansia muciniphila* and *Lactobacillus johnsonii*↓ *Lactobacillus murinus*↑	The LPS/TLR4/NF-κB signaling pathway↓ Neuroinflammation↓	Yang et al. (2024b)
			Healthy mice	Beneficial bacteria↑	Intestinal immunity Tryptophan metabolism	Qin et al. (2025)
	Fucosylated Chondroitin Sulfate	Sea cucumber body wall	MPTP-induced c57 mice	*Firmicutes*, *Staphylococcus*↓ *Bacteroidetes*, *Muribaculaceae*, *Lactobacillus*↑	Neuroinflammation↓ The NF-κB signaling pathway↓ The intestinal barrier disruption↓	Ye (2022)
	Ganoderma lucidum polysaccharide	Ganoderma lucidum (Curtis)P.Karst	MPTP-induced c57 mice	At the phylum level, *Firmicutes*↓ *Bacteroidetes*, *Actinobacteria* and *Proteobacteria*↑ at the genus level, *Lactobacillus*, *unclassified_Lachnospiraceae*, *Lachnospiraceae_NK4A136_group*, *Lachnospiraceae_UCG-006, Alloprevotella*, *Lachnoclostridium, Ruminiclostridium_9*, *Rikenella*, *Blautia* and *Ruminococcaceae_UCG-014*↑ *Aerococcus*, *Bacteroides*, *Corynebacterium_1*, *Erysipelotrichaceae*, *Erysipelatoclostridium* and *Staphylococcus*↓	The TLR4/MyD88/NF-κB signaling pathway↓ Inflammation↓	Chen et al. (2025)
	Polymannuronic acid	Brown algae	MPTP-induced c57 mice	Gut microbiota derived SCFAs↑	Inflammation↓ MAPK signaling pathway↓ The intestinal barrier and BBB disruption↓	Dong et al. (2020)
			Healthy Kunming mice	At the phylum level *Tenericutes*, *Bacteroidets*, *Firmicutes*↑ At the genus level, *Escherichia*↓ *Prevotrlla*↑	Lipid metabolism	Song (2023)
	Gastrodia elata polysaccharide	Gastrodia elataBl.	MPTP-induced c57 mice	Regulated the dysbiosis of PD-related gut microbiota such as *Akkermansia*, *Lactobacillus*, *Bacteroides*, *Prevotella*, *Faecalibacterium*	Apoptotic and inflammatory signaling pathways↓	Gan et al. (2024b)
	Arabinoxylans	Cereal grains	Rotenone-induced SD rats	*Firmicutes*, *Clostridia*, *Lachnospirates* and *Lachnospiraceae*↑	Neuroprotection↑	Luo et al. (2023)
			Rotenone-induced SD rat	*Blautia*, *Bacteroides*↑	Intestinal permeability↓ Intestinal inflammation↑	Bao (2023)
	Trehalose	Fungi, insects and plants	PrP-A53T male mice	*Lachnospiraceae* and *Ruminococcaceae*↑	Neuroprotection↑ GLP-1 secretion↑	Pradeloux et al. (2024)
Terpenoids and Glycosides	Panaxadiol	Panax species	Rotenone-induced C57 mice	*Unclassified Clostridiales*, *unidentified Clostridiales*, *Coprococcus* ↑ *Ruminococcus*↓	The TLR4/MyD88/NF-κB pathway↓ inflammation↓ The BBB damage↓	Xu et al. (2025)
	Rubusoside	Rubus chingii var. suavissimus	MPTP-induced c57 mice	*Actinobacteriota* and *Patescibacteria*↑	Neuronal apoptosis↓ Microglia activation and inflammatory response↓ Amino acid metabolism The JNK/MAPK/NF-κB pathways↓	Meng et al. (2024)
	Withaferin A	Withania somnifera	A53T transgenic mice	Anti-inflammatory gut bacteria (e.g.*Bifidobacterium*, *Dubosiella*, *Akkermansia*)↑ *Ligilactobacillus*, *Lactobacillus_murinus*↓	Sphingolipid signaling pathways Anti-inflammatory activity	Sun et al. (2025)
	Salidroside	Rhodiola spp.	MPTP-induced c57 mice	At the phylum level, *Bacteroidetes*↑ *Firmicutes*↓ At the syllabus level, *o_Bacillales*↓ *o_Bacteroidales_c_Bacteroidia*↑ At the family levels *Lactobacillaceae*, *Lachnospiraceae*↑ *Aerococcaceae*, *Staphylococcaceae*↓ At the genus level *Lactobacillus*↑ *Aerococcu*, *Desulfovibrio*, *Staphylococcus*↓	Neuroprotection↑ Activation of glial cells↓ Inflammation↓ The TAU metabolic pathway↑	Cui (2022)
	Cordycepin	Cordyceps sinensis	DSS-induced c57 mice	At the phylum level *Firmicutes*, *Desulfobacterota*, *Actinobacteriota*, *Patescibacteria*, *Fusobacteriota* and *Myxococcota*↓ *Proteobacteria*, *Campylobacterota* and *Deferribacterota*.↑ At the genus level, *Candidatus_Saccharimonas*, *Anaerotignum*, *Lactobacillus*, *HT002*↑ *Alistipes*, *Escherichia_Shigella*, *Eubacterium*↓	Imbalances in Th1/Th2 and Th17/Treg Immune axes↓	Liu et al. (2024b)
Alkaloids	Berberine	Berberidaceae and Ranunculaceae plants	ICR mice	*Enterococcus*, *Escherichia–Shigella*, *Pseudomonas* and *Lactobacillus*↑	The Phe–Tyr–dopa–dopamine metabolic pathway↑	Wang et al. (2021b)
			PD patients	Alteration of Alpha-Diversity and Beta-Diversity	Inflammation↓	Li et al. (2022a)
			6-OHDA-induced PD mice	--	Gastrointestinal dysfunction↓	Liu et al. (2025)
	Piperine	Piper nigrum and Piper longum	6-OHDA-induced PD rats	*Bacteroides* and *Prevotella*↑ *Salmonella* and *Escherichia*↓	PI3K/AKT/mTOR-mediated gut-brain autophagy	Yu et al. (2024)
			6-OHDA-induced PD rats	The gut-associated strain *Enterococcus faecalis*↓	Levodopa availability↑	Hu et al. (2024)
Others	Alpha-linolenic acid	Perilla seed oil	Rotenone-induced c57 mice	*Bifidobacteria*, *Lactobacillus* and *Faecalibacteria*↑ *Turicibacter*, *Ruminococcus* and *Akkermansia*↓	Microglia activation↓ Inflammation↓ Neuroprotection↑	Techaniyom et al. (2024)
	Sika Deer Velvet Antler Peptide	Male deer	MPTP-induced c57 mice	*Prevotellaceae*, *Helicobacteraceae*, *Prevotella*↑	The SIRT1-dependent Akt/Nrf2/HO-1-signaling pathway↑ Oxidative stress↓ Neuroprotection↑ The MAPKs/Akt pathway↓ Microglia activation and apoptosis↓	Liu et al. (2024a)
	Allantoin	Leguminous species	MPTP-induced c57 mice	*Streptococcaceae* and *Bifidobacteriales*↑ At the phylum level, *Actinobacteria*, *TM7* and *Deferribacteres*↑ At the family level, *Lactobacillaceae*↑ *Enterobacteriaceae* and *Rumiinococcaceae*↓ At the genus level, *Akkermansia* and *Lactobacillus*↑ *Oscillospira*↓	Inflammation by the NF-κB and MAPK signaling pathways↓ Oxidative stress by the AKT/Nrf2/HO-1 signaling pathway↓ Microglia activation↓	Yang et al. (2024a)

### 3.1 Phenols

#### 3.1.1 Polyphenols

##### 3.1.1.1 Resveratrol

Resveratrol (RSV), a naturally occurring polyphenolic compound, is predominantly found in jackfruit, grapes, red wine, and other sources. RSV supplementation has been demonstrated to elevate *Akkermansia*, *Bacteroide*s, and *Blautia* abundance while increasing microbial metabolite 4-HPA and activating SIRT1 signaling (Wang P. et al., 2025). SIRT1 modulates oxidative stress and mitochondrial function to counteract neurodegenerative processes, thereby protecting dopaminergic neurons from α-syn-induced toxicity and apoptosis (Thapa et al., 2024). Notably, SIRT1 reduction occurs in both PD patients and models (Singh et al., 2017).

RSV significantly restores the levels of beneficial gut bacteria/metabolites, ameliorating PD-related pathological progression and symptoms. Preclinical studies demonstrate RSV’s dual regulatory actions: it modulates dopamine receptor transcription and calcium homeostasis-related genes while downregulating ZO-1/occludin expression. This molecular remodeling reconstructs the intestinal barrier. Consequently, colonic α-syn accumulation diminishes with concomitant reduction of low-grade inflammation (Sun et al., 2024a). Furthermore, FMT from RSV-treated mice markedly alleviates PD phenotypes in recipient mice and increases TH-positive cells in the SN and striatal TH-positive fiber density (Tao et al., 2023). Additionally, RSV reduces LPS, inhibits the TLR4/MyD88/NF-κB signaling pathway-mediated inflammation, repairs the IB, and modulates the gut-brain axis to exert neuroprotective effects (Gui et al., 2024). Through these multifaceted mechanisms targeting the microbiota-gut-brain axis, RSV demonstrates significant therapeutic potential for PD treatment.

##### 3.1.1.2 Curcumin

Curcumin (Cur) is a bioactive low-molecular-weight polyphenolic compound primarily extracted from the rhizomes of Curcuma longa. It exhibits broad pharmacological effects, including anti-inflammatory, antioxidant, and neuroprotective properties (Wang G. et al., 2025). Clinical studies confirm its neuroprotective efficacy (Chang, 2025). Crucially, Cui et al. identify GM and metabolites as primary mediators of these effects (Cui et al., 2022). Cur alleviates gastrointestinal dysmotility and motor deficits in PD by modulating GM dysbiosis. This modulation suppresses inflammatory responses, leading to three key outcomes: 1) reduced α-syn aggregation; 2) enhanced IB integrity; 3) protection of dopaminergic neurons in SN. At the molecular level, preclinical studies demonstrate Cur’s activation of the SIRT1/NRF2 pathway. This activation inhibits AIM2-mediated pyroptosis and caspase-1. Simultaneously, Cur suppresses NF-κB and NLRP3 pathways. Consequently, proinflammatory cytokine production decreases while TH levels elevate in the SN and striatum (Zhong et al., 2022; Li W. et al., 2025). Furthermore, Cur enriches SCFA-producing genera like *Turicimonas* and *Culturomica* (Cai et al., 2023). This enrichment enhances carbohydrate metabolism and gastrointestinal motility. PD progression is thereby delayed. Additionally, Cur scavenges free radicals effectively. It concurrently suppresses neuroinflammation and oxidative stress while restoring mitochondrial function. These findings validate Cur’s multi-target therapeutic action against PD via the GMBA.

##### 3.1.1.3 Chicoric acid

Chicoric acid (CA) is a core polyphenolic active component found in medicinal plants such as chicory, dandelion, and Echinacea. It exhibits anti-inflammatory and neuroprotective pharmacological effects and has a long history of clinical application (Yang et al., 2022). Recent studies have demonstrated that CA can modulate neuroinflammation and neurodegenerative pathology in PD through multi-target regulation of the gut-brain axis (Wang et al., 2022b). Specifically, Wang et al. confirmed that oral administration of CA reshapes GM balance and elevates SCFAs levels. Furthermore, CA restores intestinal epithelial integrity and suppresses the TLR4/MyD88/NF-κB inflammatory signaling pathway (Wang et al., 2022a). This cascade of actions blocks the gut-brain axis inflammatory cascade, thereby significantly improving motor dysfunction in PD.

##### 3.1.1.4 Eigallocatechin-3-gallate

Eigallocatechin-3-gallate (EGCG), primarily derived from green tea, demonstrates neuroprotective properties. Epidemiological evidence indicates that consuming over 600 mL of green tea daily correlates with reduced PD risk (Hu et al., 2007). Preclinical PD studies have established EGCG’s efficacy in mitigating neurodegeneration in the SN by targeting pathogenic protein misfolding and aggregation (Gonçalves et al., 2021; Sergi, 2022). Notably, EGCG’s protective mechanisms involve modulation of the GM. As demonstrated by Xu et al., EGCG restructures GM composition and ameliorates neuronal/mitochondrial morphology in *Drosophila* PD models via the TotM pathway—a mechanism associated with mitochondrial function (Xu et al., 2020). Additionally, EGCG repairs compromised IB integrity and attenuates inflammatory responses (Li S. et al., 2024). Crucially, GM metabolizes EGCG into bioactive metabolites with enhanced BBB permeability (Pervin et al., 2019). Collectively, these findings indicate that GM serves as a pivotal hub mediating EGCG’s neuroprotective effects through metabolic conversion.

#### 3.1.2 Flavonoids

##### 3.1.2.1 Typha pollen flavonoids

Typhae Pollen Flavonoids (TPF), natural compounds extracted from the pollen of the traditional medicine Typha angustifolia, represent the core bioactive constituents responsible for its pharmacological effects in promoting blood circulation, resolving stasis, and hemostasis. In MPTP-induced PD models, TPF significantly restructures GM composition and ameliorate dysbiosis, evidenced by reduced pathogenic bacterial abundance and increased beneficial taxa. The regulatory mechanisms involve three primary pathways: 1) IB restoration: upregulation of tight junction protein occludin expression; 2) Microbial metabolite modulation: Enhanced SCFAs production activates the AMPK signaling pathway, thereby suppressing neuronal ferroptosis; 3) Amino acid metabolic reprogramming. Collectively, these effects attenuate oxidative stress in the SN and inhibit pathological α-syn aggregation (Wang, 2024). Our findings demonstrate that TAF protects dopaminergic neurons via the MGBA, ultimately improving motor dysfunction in PD mice.

##### 3.1.2.2 Anthocyanin

Anthocyanins, a class of water-soluble flavonoid pigments ubiquitously present in vacuoles of flowers, fruits, stems, leaves, and roots of higher plants, are renowned for their antioxidant properties. Preclinical studies indicate that anthocyanins confer neuroprotective effects against PD (Zaa et al., 2023). In MPTP-induced PD models, anthocyanins paradoxically reduce GM α-diversity while modulating the *Firmicutes*/*Bacteroidetes* ratio, ameliorating microbial dysbiosis (Gao, 2022; Wang W. et al., 2022; Cao et al., 2024). This restructuring attenuates dopaminergic neuron damage and motor deficits via the gut-brain axis. Mechanistically, anthocyanins: downregulate pro-inflammatory cytokines (TNF-α, IL-1β, IL-6) and increase TH-positive neurons in the SN (Wang Y. et al., 2025). In addition, anthocyanins activate the Nrf2/GPX7 signaling pathway to mitigate mitochondrial oxidative stress, thereby restoring mitochondrial function and reducing neurodegeneration (Li X. N. et al., 2025).

##### 3.1.2.3 Daidzein

Daidzein (DAI), dietary phytochemicals naturally occurring in soybeans and their derivatives, demonstrate ameliorative effects in neurodegenerative disorders. Following GM-mediated conversion to equol (EQL)—a metabolite with superior BBB permeability—this active compound accumulates in the CNS (Shimazu et al., 2021). Studies confirm that EQL significantly attenuates neurotoxicity and inflammation in both cellular and animal models of neurotoxin-induced PD (Johnson et al., 2020). Mechanistically, EQL suppresses microglial hyperactivation via inhibiting the TLR4/MAPK/NF-κB inflammatory cascade. Meanwhile, EQL enhances production of neurotrophic factors (e.g., BDNF, GDNF) in astrocytes. These coordinated actions collectively reduce neuronal apoptosis and confer resistance against inflammatory damage (Subedi et al., 2017). Our findings establish that the neuroprotection afforded by DAI critically depends on the anti-inflammatory mechanisms of its GM-derived metabolite EQL.

##### 3.1.2.4 Ginkgolide C

Ginkgolide C (GC), a terpene lactone isolated from the traditional herbal medicine Ginkgo biloba leaves, exhibits anti-inflammatory, antioxidant, and free radical-scavenging properties with demonstrated BBB permeability for neuroprotection. Gao et al. revealed that oral GC administration significantly ameliorates MPTP-induced neurodegeneration in PD models, mechanistically linked to GM modulation—particularly through normalization of the *Firmicutes*/*Bacteroidetes* ratio (Gao et al., 2024). Further evidence indicates GC preserves IB integrity via activating the AKT/Nrf2/HO-1 antioxidant axis and suppresses NF-κB/MAPK inflammatory signaling cascades. Collectively, these actions attenuate gut-brain axis dysregulation, establishing GC as a dual-target therapeutic agent that concurrently mitigates oxidative stress and neuroinflammation to delay PD progression (Xu D. et al., 2022).

##### 3.1.2.5 Brazilin

Brazilin is a characteristic homoisoflavonoid compound derived from the heartwood of *Caesalpinia sappan L.* (Leguminosae), mediates this plant’s traditional medicinal effects in promoting blood circulation, resolving stasis, and alleviating pain/swelling. Recent studies in PD have demonstrated that brazilin inhibits α-syn aggregation and ameliorates motor dysfunction. These findings position brazilin as a novel therapeutic candidate for PD (Cui Z. et al., 2024). Gao et al. reported that brazilin modulates GM to enhance butyrate production. This microbial metabolite upregulates the expression of ZO-1 and occludin to mitigate IB damage. Simultaneously, butyrate attenuates systemic inflammatorion and dopaminergic neurodegeneration (Gao et al., 2025). Butyrate has been shown to inhibit M1-like polarization of colonic macrophages and the production of pro-inflammatory cytokines, including TNF-α and IL-1β, via the PPARα-CYP4X1 axis (Chen Y. et al., 2022).

##### 3.1.2.6 Neohesperidin

Neohesperidin (Neo), a natural dihydroflavonoid glycoside primarily derived from *Poncirus* and *Citrus* genera (Rutaceae family), exhibits notable anti-inflammatory activity. In an MPTP-induced PD mouse model, He et al. demonstrated that oral Neo administration upregulates the abundance of *Prevotella* and *Bacteroides* (He et al., 2024). *Prevotella* promotes dietary fiber fermentation to produce SCFAs, whereas *Bacteroides* enhances mucus layer integrity—mechanisms that collectively maintain IB homeostasis (Yu et al., 2023). Molecular analyses further confirmed SCFAs upregulate ZO-1 and occludin expression. These findings indicate Neo restores IB integrity via GM modulation. Additionally, their study revealed Neo suppresses NF-κB and MAPK signaling pathways, thereby reducing pro-inflammatory cytokines (TNF-α, IL-1β) in both intestinal and cerebral tissues. This dual inhibition protects dopaminergic neurons in the SN and mitigates neurodegeneration. Collectively, Neo exerts neuroprotective effects through synergistic modulation of GM and suppression of inflammatory cascades.

##### 3.1.2.7 Fisetin

Fisetin, a naturally occurring flavonoid abundant in fruits (e.g., strawberries, apples), exhibits multi-targeted neuroprotective properties encompassing antioxidant, anti-inflammatory, and immunomodulatory activities (Calabrese et al., 2025). Recent studies have revealed an association between fisetin’s neuroprotective properties and the GM. Chen et al. demonstrated that fisetin intervention in MPTP-induced mice resulted in a significant increase in the abundance of *Lachnospiraceae*, accompanied by marked reductions in *Escherichia-Shigella* and *Bacillus* abundance (Chen et al., 2020). *Lachnospiraceae* promotes butyrate production, which exerts anti-inflammatory effects (Guo et al., 2024). These findings suggest that fisetin alleviates neurodegeneration and delays PD progression by remodeling the GM.

#### 3.1.3 Alkylphenols

##### 3.1.3.1 6-shogaol

6-Shogaol (6S), primarily derived from the dried rhizomes of the perennial herb Zingiber officinale, exhibits anti-inflammatory, antioxidant, barrier-repairing, and neuroprotective activities. Numerous studies have demonstrated the significant potential of 6S in the treatment of neurodegenerative diseases. It has been shown to modulate MPTP-induced intestinal inflammatory responses, intestinal barrier integrity, and enteric neuronal dysfunction (Huh et al., 2020). Eugene Huh et al. reported that 6S inhibited *Proteus mirabilis*, ameliorating intestinal barrier disruption, motor dysfunction, and neuronal death (Huh et al., 2023b).

### 3.2 Polysaccharides

#### 3.2.1 Chitosan

Chitosan is primarily derived from the exoskeletons of crustaceans, mollusks, and fungal cell walls. Chitosan exerts neuroprotective effects through mechanisms including free radical scavenging, antioxidation, inhibition of apoptosis, and anti-inflammatory actions (Zhu et al., 2024). Preclinical studies demonstrate that chitosan enhances, ETC enzyme activity, restores dopamine levels, and ameliorates motor dysfunction and neurotoxicity (Pramod Kumar and Harish Prashanth, 2020). These actions establish its neuroprotective efficacy via mitochondrial functional restoration. Furthermore, chitosan crosses the BBB to suppress neuroinflammation through TSPO/c-Fos pathway-mediated downregulation of TNF-α and iNOS. This mechanism protects dopaminergic neurons from inflammatory damage (Chen, 2024). Moreover, emerging evidence indicates chitosan modulates gut-brain crosstalk by reducing acetate levels and repairing intestinal and BBB impairments (Wang et al., 2026). These dual-barrier interventions suggest microbial metabolite regulation contributes to PD pathology mitigation. Notably, the specific effects on GM composition and microbial metabolites remain unverified—a key limitation requiring further investigation.

##### 3.2.1.1 Fucoidan

Fucoidan, a sulfated polysaccharide abundantly present in the mucilage of brown algae (e.g., *Laminaria*, *Undaria pinnatifida*, *Fucus*, and *Nemacystus decipiens*), exhibits potent immunomodulatory properties with emerging neuroprotective applications in PD via the MGBA. Preclinical evidence demonstrates that fucoidan remodels GM composition and suppresses LPS/TLR4/NF-κB signaling to modulate microglial polarization in SN (M1-type markers TNF-α and iNOS reduction, with M2-type markers Arg-1 and CD206 elevation) (Yang X. et al., 2024). Mechanistically, fucoidan enhances macrophage autophagy activity, amplifying anti-inflammatory responses (Hou et al., 2025). Fucoidan also regulates tryptophan metabolism via microbial modulation (kynurenine pathway) and strengthens IB integrity (Qin et al., 2025; Xue et al., 2025). Collectively, fucoidan coordinates gut-immune-brain crosstalk to mitigate PD pathogenesis through synergistic microbiota modulation and immunoregulation.

##### 3.2.1.2 Fucosylated chondroitin sulfate

Fucosylated Chondroitin Sulfate (FuCS), a biologically active glycosaminoglycan (GAG) extracted from sea cucumber body wall. Ye’s study demonstrated that FuCS modulated the GM by reducing the abundance of the conditionally pathogenic bacterium *Staphylococcus* while increasing probiotic *Muribaculaceae*. Concomitantly, FuCS decreased the expression of *Soyasapogenol E*—a metabolite positively correlated with IL-1β—and lowered α-syn levels. These findings indicate that FuCS alleviates nigral neurodegeneration and motor dysfunction in PD mice through GM-mediated anti-inflammatory effects (Ye, 2022). Furthermore, FuCS enhanced IB function by upregulating colonic expression of tight junction proteins ZO-1, occludin, and claudin-1.

##### 3.2.1.3 Ganoderma lucidum polysaccharide

Ganoderma lucidum Polysaccharide (GLP) is a naturally occurring polysaccharide primarily derived from the fruiting bodies, mycelia, or spores of the medicinal fungus *Ganoderma lucidum (Curtis) P. Karst*. Pharmacological studies have demonstrated that GLP exhibits anti-inflammatory, antioxidative, and anti-apoptotic effects (Liu et al., 2023). Recent studies demonstrate that GLP reduces α-syn expression in PD models while modulating GM abundance, diversity, and evenness. GLP concurrently elevates SCFA levels and suppresses the intestinal TLR4/MyD88/NF-κB pathway, thereby downregulating pro-inflammatory cytokines including IL-6 and IL-1β (Chen et al., 2025). These findings indicate that GLP exerts neuroprotective effects by mitigating inflammation through GM regulation and microbial metabolites-SCFAs.

##### 3.2.1.4 Polymannuronic acid

Polymannuronic acid (PM), an oligosaccharide derived from brown algal alginate, is a structural polysaccharide exhibiting diverse biological activities—including antioxidant, immunomodulatory, antihypertensive, hypoglycemic, neuroprotective, and antimicrobial effects. Song’s research revealed that low-concentration PM significantly reduces the abundance of the opportunistic pathogen *Escherichia* while increasing the prevalence of the beneficial genus *Prevotella* in the GM (Song, 2023). Further studies indicate that PM modulates GM in PD, elevating SCFA levels to mediate neuroprotective effects via the gut-brain axis (Dong et al., 2020). Simultaneously, PM suppresses the MAPK pathway, mitigating systemic inflammation and enhancing the integrity of both IB and BBB.

##### 3.2.1.5 Gastrodia elata polysaccharide

Gastrodia elata polysaccharide (GEP), derived from dried tubers of the orchidaceous plant *Gastrodia elata Bl,* is believed in traditional medicine to possess therapeutic effects such as calming endogenous wind, relieving convulsions, and suppressing hyperactive liver yang. Mechanistically, GEP modulates gut-brain axis activity to support neurological health (Gan Q. et al., 2024). As demonstrated by Gan et al., GEP ameliorates GM dysbiosis (notably enhancing *Akkermansia* and *Lactobacillus* abundance) while elevating SCFA levels (Gan Q. X. et al., 2024). Concomitantly, it upregulates occludin expression and reduces pro-inflammatory cytokines (TNF-α, IL-1β, IL-6), collectively inhibiting α-syn accumulation and dopaminergic neuron loss. These findings indicate GEP exerts neuroprotective effects in PD mice by restoring the IB and mitigating inflammation via GM.

##### 3.2.1.6 Arabinoxylans

Arabinoxylan (AX), a non-starch polysaccharide primarily found in the cell walls of cereal grains and other plants, exhibits immunomodulatory activity. Preclinical evidence in PD indicates that AX increases the relative abundance of *Firmicutes* and *Clostridia*, elevates TH levels in the SN, and reduces α-syn accumulation (Luo et al., 2023). These findings suggest that AX exerts neuroprotective effects via modulation of the GM. Further studies by Bao revealed that AX promotes the growth of microbiota negatively associated with PD, enhances SCFA production, and increases GLP-1 secretion by 29% (Bao, 2023). This indicates that the neuroprotective effects of AX in PD are primarily mediated indirectly through the GM and GLP-1.

##### 3.2.1.7 Trehalose

Trehalose, a non-reducing disaccharide composed of two glucose molecules, is primarily found in fungi, insects, plants, and certain animals. It exhibits biological activities, including antioxidant and neuroprotective effects. In a study by Pradeloux et al. using PrP-A53T transgenic mice as a model, the effects of trehalose on GM were investigated. The results demonstrated that trehalose increased *Lachnospiraceae* abundance, elevated GLP-1 expression, and reduced THR levels in both brain and gut tissues, thereby exerting neuroprotective effects (Pradeloux et al., 2024).

### 3.3 Terpenoids and glycosides

#### 3.3.1 Panaxadiol

Panaxadiol, a naturally derived bioactive compound extracted from Panax species of the Araliaceae family, belongs to the dammarane-type triterpenoid sapogenins and exhibits multi-target regulatory capabilities, particularly in neuroprotection, cardiovascular protection, anti-inflammatory effects, and antioxidant activity. The highest concentration of panaxadiol is observed in the gastrointestinal tract of rats, where it exerts anti-inflammatory effects on macrophages by modulating the MAPK pathway, specifically through suppressing the phosphorylation (activation) of p38 and ERK (Xu L. et al., 2022). In a study by Xu et al., the impact of panaxadiol on PD was investigated at both cellular and animal levels (Xu et al., 2025). It was demonstrated for the first time that panaxadiol significantly restores GM composition, suppresses downstream pro-inflammatory cytokine production via the TLR4/MyD88/NF-κB pathway, reduces activation of SN glial cells, and attenuates peripheral and central inflammation. Additionally, it mitigates BBB damage by upregulating tight junction proteins (ZO-1, occludin, and claudin-5), repairs neuronal loss, and improves motor and gastrointestinal dysfunction.

#### 3.3.2 Rubusoside

Rubusoside (Ru), a diterpene glycoside from the leaves of *Rubus chingii* var. *suavissimus*, is a high-intensity, low-calorie natural sweetener with potent anti-inflammatory properties. Preclinical evidence indicates that Ru alleviates LPS-induced low-grade chronic inflammation (Zhang et al., 2020). Ru has been demonstrated to modify the composition of gut microbiota and metabolite profiles (particularly amino acid metabolism) in PD model mice, inhibit microglial activation to reduce the release of proinflammatory mediators, regulate the JNK/p38 MAPK/NF-κB signaling pathway, mitigate oxidative stress, prevent neuronal apoptosis, and protect dopaminergic neurons while improving motor dysfunction (Meng et al., 2024). These findings suggest the potential of Ru in delaying PD progression through modulation of GM.

#### 3.3.3 Withaferin A

Withaferin A (WFA) is a naturally occurring steroidal lactone compound with diverse biological activities, including anti-inflammatory, antiviral, and neuroprotective effects. It is primarily derived from the leaves of *Withania somnifera*. Sun et al. administered WFA to A53T transgenic mice (Sun et al., 2025), observing an increase in the abundance of anti-inflammatory GM and treversed alterations in 55 metabolites associated with sphingolipid metabolism, dopaminergic synapses, and neuroactive ligand-receptor interactions. These findings demonstrate that WFA modulates sphingolipid signaling pathways within the MGBA, mitigates neuroinflammation, and promotes neuronal repair.

#### 3.3.4 Salidroside

Salidroside (SAL), a phenylethanoid glycoside from *Rhodiola* spp., exhibits multifaceted pharmacological activities, including GM modulation, antioxidative, anti-inflammatory, and neuroprotective effects. Cui et al. demonstrated that SAL intervention in PD models increased *Lactobacillus* abundance while reducing *Aerococcus*, *Desulfovibrio*, and *Staphylococcus*; modulated TAU-associated metabolic pathways; enhanced intestinal length and structural integrity; downregulated IL-6, IL-1β, and TNF-α expression; inhibited α-syn deposition and glial activation; and ameliorated motor dysfunction and neuronal loss (Cui, 2022).

#### 3.3.5 Cordycepin

Cordycepin, a nucleoside compound derived from the medicinal fungus *Cordyceps* sinensis, demonstrates significant therapeutic potential against neuroinflammation in PD by targeting multiple pathways, including NLRP3 inflammasome and TLR/NF-κB signaling (Cheng and Zhu, 2019; Zhang et al., 2021; Sharma et al., 2023). Research by Liu et al. revealed cordycepin’s dual action: improving GM composition while modulating the Th1/Th2 and Th17/Treg immune axis balance (Liu Z. et al., 2024). Crucially, cordycepin suppresses PI3K/AKT/mTOR and ERK/JNK pathway activation, alleviates neuroinflammation, and enhances autophagy-related protein expression in the striatum and SN. This cascade ultimately reduces neuronal apoptosis in MPTP-induced PD mice (Wang L. et al., 2024). Furthermore, cordycepin regulates adenosine A2A receptors and ameliorates cognitive dysfunction in PD models, offering a novel therapeutic strategy for PD-associated dementia (Huang et al., 2023).

### 3.4 Alkaloids

#### 3.4.1 Berberine

Berberine (BBR), an isoquinoline alkaloid primarily biosynthesized by Berberidaceae (e.g., *Berberis* spp.) and Ranunculaceae (e.g., *Coptis* and *Thalictrum* spp.) plants, exerts neuroprotective effects by crossing the BBB (Begh et al., 2025). Clinical evidence indicates that oral BBR administration activates the gut-brain axis, promoting levodopa production by *Enterococcus* to increase cerebral dopamine levels (Wang Y. et al., 2021). Li et al. demonstrated BBR’s efficacy in PD patients by improving GM dysbiosis and suppressing inflammatory cytokines (Li J. et al., 2022). In PD rat models, BBR ameliorated colonic dysfunction, restored mucosal permeability, modulated intestinal neurotransmitters and EGCs, and alleviated depressive-like behaviors (Liu et al., 2025). Collectively, these findings establish GM as a pivotal mediator of BBR’s neuroprotective effects.

#### 3.4.2 Piperine

Piperine (PIP) is a pungent cinnamoyl amide alkaloid extracted from the fruits of *Piper nigrum* and *Piper longum*. As a medicinal and edible compound, it exhibits anti-inflammatory, antioxidant, cognitive-enhancing, gastrointestinal-protective, and antidepressant properties. Clinical evidence indicates that PIP modulates GM by upregulating *Bacteroides* and *Prevotella* while downregulating *Salmonella* and *Escherichia*. It elevates SCFA levels, restores IB integrity, and alleviates neuronal loss and α-Syn aggregation. These effects occur through gut-brain axis autophagy modulation via the PI3K/AKT/mTOR pathway, ultimately improving motor deficits and gastrointestinal dysfunction in PD rat models (Yu et al., 2024). Additionally, Hu et al. demonstrated that PIP enhances L-dopa bioavailability and increases cerebral dopamine by suppressing *Enterococcus faecalis*-mediated L-dopa metabolism in the gut, thereby ameliorating motor impairments in PD rats (Hu et al., 2024).

### 3.5 Others

#### 3.5.1 Alpha-linolenic acid

Alpha-linolenic acid (ALA), an essential omega-3 polyunsaturated fatty acid, is abundant in perilla seed oil and constitutes 51%–64% of its composition. With potent antioxidant and anti-inflammatory properties, ALA exerts neuroprotective effects through gut-brain axis modulation (Mahnoor et al., 2025). Peerapa et al. demonstrated that ALA enhances GM diversity and boosts SCFA production. Concurrently, it reduces α-syn accumulation in both colonic myenteric plexus and hippocampal regions, suppresses microglial activation, attenuates inflammatory cascades, and increases colon tissue length (Techaniyom et al., 2024). Furthermore, ALA upregulates TH expression in SN and striatal regions while ameliorating motor and non-motor symptoms. These findings establish ALA as a multi-mechanism modulator of GM-mediated neuroprotection.

#### 3.5.2 Sika deer velvet antler peptide

Sika Deer Velvet Antler Peptide (VAP) is derived from the non-ossified antlers of male deer, exhibiting tonic properties and immunomodulatory effects. Peptidomic analysis has identified 189 peptides with notable metal-binding characteristics. Studies have demonstrated that VAP increases the abundance of *Prevotellaceae*, *Helicobacteraceae*, and *Prevotella* while activating the SIRT1-mediated Akt/Nrf2/HO-1 pathway to mitigate MPP-induced apoptosis and oxidative stress (Liu Y. et al., 2024). Furthermore, it suppresses microglial activation and reduces the phosphorylation levels of the MAPK/Akt inflammatory pathway, subsequently lowering the expression of inflammatory mediators and preventing neuroinflammation. These findings suggest that VAP possesses neuroprotective potential in PD.

#### 3.5.3 Allantoin

Allantoin, a naturally occurring compound predominantly found in plants (notably leguminous species), exhibits anti-inflammatory, antioxidant, and soothing properties. As demonstrated by Shuo Yang et al., allantoin modulates the GM, exerts antioxidative effects via the AKT/Nrf2/HO-1 signaling pathway, and suppresses inflammatory responses through inhibition of the NF-κB and MAPK signaling pathways (Yang S. et al., 2024). Additionally, it contributes to intestinal barrier restoration and alleviates neurodegeneration.

## 4 Limitations in the clinical translation of natural products

Current research on natural product-mediated GM modulation for PD treatment remains predominantly preclinical, lacking large-scale clinical evidence. Existing clinical data demonstrate that FMT significantly improves autonomic symptoms in PD patients, while probiotic therapy markedly reduces disease severity, anxiety, and gastrointestinal disturbances (Cheng et al., 2023; Andreozzi et al., 2024; Bruggeman et al., 2024; Zali et al., 2024). These findings suggest the potential of GM modulation in PD prevention and treatment. These findings highlight GM’s therapeutic potential for PD. Traditional medicine studies confirm that *Chaihu Jia Longgu Muli* decoction regulates GM and decreases oxidative stress in PD patients (Zhang S. et al., 2024).

However, clinical translation of natural products faces key limitations. The inherent complexity of natural products poses challenges in standardization. Poor solubility, structural instability, rapid metabolism, and low bioavailability hinder their clinical application. Additionally, pharmacokinetic challenges prevent these compounds from reaching effective concentrations in target tissues. The toxic side effects associated with natural products remain insufficiently studied, necessitating further toxicological evaluations. Furthermore, clinical trial design presents difficulties. Identifying sensitive and specific clinical endpoints and biomarkers presents a major challenge. However, these markers must reflect actual disease modification or microbiota modulation—not merely symptomatic relief.

## 5 Discussion and conclusion

As the second most prevalent neurodegenerative disorder, PD has been extensively linked to the GM, with mounting evidence suggesting a profound association between microbial communities and the interindividual variability in PD severity and clinical manifestations. Research on PD pathophysiology and therapeutic strategies has shifted from the traditional brain-centric paradigm to a novel perspective centered on the MGBA. Natural products, owing to their multi-target effects and low toxicity, exhibit unique advantages in modulating the MGBA. Although some natural products suffer from limitations such as poor bioavailability and limited BBB permeability, existing studies have demonstrated that their interactions with GM constitute a sophisticated “biotransformation-signal amplification” system, exerting direct or indirect therapeutic effects against PD.

In recent years, significant breakthroughs have been made in elucidating the regulatory role of natural products in PD treatment via GM modulation. This review systematically summarizes natural products that mediate GM-based PD therapy and their underlying mechanisms. Polyphenols, flavonoids, polysaccharides, alkaloids, terpenoids, and glycosides exert anti-PD effects by modulating the diversity and abundance of PD-associated GM, altering metabolite levels, inhibiting α-Syn pathological aggregation, improving barrier function, and mitigating neuroinflammation, mitochondrial dysfunction, and oxidative stress, thereby influencing immune, endocrine, and nervous system pathways. In multiple aforementioned preclinical studies (e.g., EGCG and Neo), the addition of antibiotics was found to attenuate the neuroprotective effects against PD, suggesting to some extent that alterations in the GM precede therapeutic efficacy. Notably, the vast diversity of GM enables the “personalized processing” of natural products, allowing these metabolites to serve as key players across multiple mechanistic pathways. Notably, although *Akkermansia* is enriched in the gut of PD patients, accumulating evidence indicates its neuroprotective potential. This apparent paradox highlights the bacterium’s environmentally modulated pleiotropy within the gut–brain axis. During homeostasis or compensatory states, it confers neuroprotection via immunomodulation and bioactive metabolite secretion. Conversely, under PD-associated chronic inflammation with IB disruption, its mucolytic activity may drive pathogenesis.

With deepening insights into the GM-PD relationship, microbiota-based therapeutic strategies hold broad clinical potential. Modulating GM may enhance patient responsiveness to conventional treatments, thereby improving therapeutic outcomes. Interdisciplinary research integrating nutriology and microbiology will provide novel perspectives for personalized PD therapy. Building on existing findings, future research faces several challenges: 1) elucidating mechanistic details and identifying therapeutic targets; 2) developing targeted delivery systems, such as nanocarriers or engineered bacterial shells, to achieve colon-specific release of natural products; 3) exploring combination therapies with natural products; 4) constructing multi-level intervention strategies; and 5) investigating pharmacokinetics, optimal dosing, and metabolic duration. Addressing these challenges remains a complex and demanding task.

In summary, natural products represent a promising avenue for PD intervention through GM modulation. With the advancement of the NIH-sponsored gut-brain axis research initiative and the maturation of personalized microbiota-based therapies, early prevention and treatment strategies targeting the gut may redefine PD clinical management, offering a means to delay the progression of this neurodegenerative disorder at its root.
